# Increasing the complexity of chromatin: functionally distinct roles for replication-dependent histone H2A isoforms in cell proliferation and carcinogenesis

**DOI:** 10.1093/nar/gkt736

**Published:** 2013-08-16

**Authors:** Rajbir Singh, Amir Mortazavi, Kelly H. Telu, Prabakaran Nagarajan, David M. Lucas, Jennifer M. Thomas-Ahner, Steven K. Clinton, John C. Byrd, Michael A. Freitas, Mark R. Parthun

**Affiliations:** ^1^Department of Molecular and Cellular Biochemistry, The Ohio State University, Columbus, OH 43210, USA, ^2^Department of Internal Medicine, The Ohio State University, Columbus, OH 43210, USA, ^3^Department of Chemistry and Biochemistry, The Ohio State University, Columbus, OH 43210, USA, ^4^Department of Molecular Virology, Immunology and Medical Genetics, The Ohio State University, Columbus, OH 43210, USA

## Abstract

Replication-dependent histones are encoded by multigene families found in several large clusters in the human genome and are thought to be functionally redundant. However, the abundance of specific replication-dependent isoforms of histone H2A is altered in patients with chronic lymphocytic leukemia. Similar changes in the abundance of H2A isoforms are also associated with the proliferation and tumorigenicity of bladder cancer cells. To determine whether these H2A isoforms can perform distinct functions, expression of several H2A isoforms was reduced by siRNA knockdown. Reduced expression of the HIST1H2AC locus leads to increased rates of cell proliferation and tumorigenicity. We also observe that regulation of replication-dependent histone H2A expression can occur on a gene-specific level. Specific replication-dependent histone H2A genes are either up- or downregulated in chronic lymphocytic leukemia tumor tissue samples. In addition, discreet elements are identified in the 5′ untranslated region of the HIST1H2AC locus that confer translational repression. Taken together, these results indicate that replication-dependent histone isoforms can possess distinct cellular functions and that regulation of these isoforms may play a role in carcinogenesis.

## INTRODUCTION

At the most fundamental level, chromatin is composed of a repeated structure known as the nucleosome. Each nucleosome consists of ∼147 base pairs of DNA wrapped around a protein complex called the histone octamer that contains two molecules of each of the four core histones (H2A, H2B, H3 and H4). The importance of chromatin structure for the packaging and regulation of eukaryotic genomes is evidenced by the extraordinary conservation of this structure throughout eukaryotic evolution. The core histones are among the most highly conserved eukaryotic proteins with many residues being completely invariant (1). However, despite this seeming uniformity, one of the most important characteristics of chromatin structure is complexity, which is necessary for encoding all of the regulatory information necessary for the proper execution of nuclear processes and for epigenetic inheritance.

The complexity of chromatin is derived from two main sources; the post-translational modification of histones and the presence of histone variants. Histones are subject to multiple forms of post-translational modification (2). Further complexity is derived from the fact that the cellular complement of most histones is not homogeneous but, rather, is composed of multiple primary sequence variants (3–5).

Histone variants can be distinguished on a number of levels. The first is the distinction between replication-dependent and replication-independent histones. Replication-dependent histones become highly expressed just before S-phase and are then repressed at the completion of DNA replication (6). Interestingly, the DNA replication-dependent histone genes are found in several large clusters that contain dozens of histone genes, and they are the only protein-coding mRNAs produced in mammalian cells that lack a poly(A) tail. Instead of a poly(A) tail, these messages contain a short highly conserved stem–loop structure in their 3′ untranslated region (UTR), and their processing and stability are regulated by the stem–loop binding protein, which specifically interacts with this structure (7,8). The DNA replication-dependent histones are used for the assembly of chromatin structure during DNA replication. Hence, the packaging of genomic DNA with the DNA replication-dependent histones is the ‘ground state’ at which chromatin structure begins.

There are also a large number of core histone genes that are constitutively expressed throughout the cell cycle and, hence, are known as replication-independent histone variants. The replication-independent histones differ in primary sequence from the replication-dependent histones with these variations ranging from only a handful of amino acid changes to the incorporation of large non-histone domains. Well-characterized examples of replication-independent histone variants include histones H3.3, H2AX, H2AZ and macroH2A (5). In addition to changes in protein sequence, the replication-independent histone genes also differ from their replication-dependent counterparts in that they are found as single genes dispersed throughout the genome, and they generate transcripts with normal poly(A) tails.

Although the replication-dependent core histones are considered to be the ‘canonical’ histones, there is actually a wide range of primary sequence variations within this group (8). To distinguish these histone variants from the replication-independent histone variants, they will be referred to here as histone isoforms. Each of the replication-dependent histones is encoded by multiple genes (H2A, 16 genes; H2B, 22 genes; H3, 14 genes; H4, 14 genes; and H1, 6 genes). Hence, the presence of distinct replication-dependent histone isoforms has the potential to significantly expand the complexity of mammalian chromatin structure. However, these core histone isoforms have not been studied in detail, as they have been presumed to encode functionally equivalent molecules.

**Figure 1.**
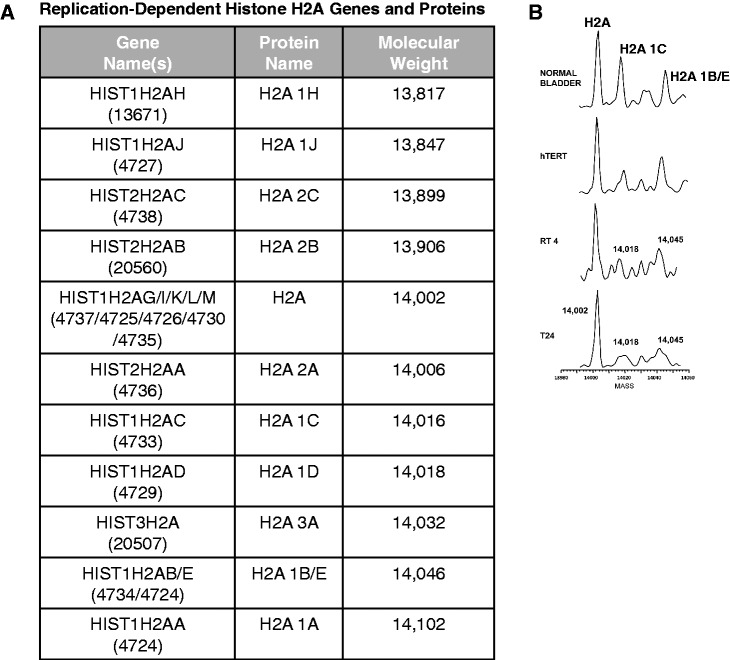
Replication-dependent histone H2A isoforms are altered in bladder cancer cells. (**A**) The table lists the genes encoding replication-dependent histone H2A isoforms along with nomenclature used to name their respective proteins and the molecular weight of each isoform. The Hugo Gene Naming Consortium (HGNC) identification number is given in parentheses. (**B**) LC/MS analysis of histone H2A isolated from the indicated cells. Normal bladder is normal human bladder epithelial cells; hTERT is immortalized human bladder epithelial cells; RT4 is non-invasive bladder cancer cells; and T24 is invasive bladder cancer cells.

Mass spectrometry-based analysis of the histone H2A complement in HeLa cells indicated that the most abundant replication-dependent isoforms were the products of several specific genes (9). The most abundant form is encoded by five distinct genes (HIST1H2AG, HIST1H2AI, HIST1H2AK, HIST1H2AL and HIST1H2A, see Figure 1A). The second most abundant species is encoded by a single gene, HIST1H2AC, and the third most abundant species is encoded by two genes, HIST1H2AB and HIST1H2AE. The nomenclature that has developed to describe these histone variants has not systematically addressed the naming of these replication-dependent histone isoforms (8,10). Therefore, they will be referred to using a nomenclature that is based on the more systematic naming of the genes that encode these proteins. The names of the replication-dependent histone genes provide important information about the location of the gene. The first part of the gene name refers to the histone gene cluster in which it resides (i.e. HIST1, HIST2 or HIST3). The next part of the name refers to the specific type of histone encoded by the gene (i.e. H1, H2A, H2B, H3 or H4). Finally, the multiple copies of each histone type are designated alphabetically based on their order within each cluster (centromere distal to proximal). Therefore, HIST1H2AC refers to the third histone H2A gene present in histone cluster 1. In referring to the H2A protein isoforms, the most abundant form of H2A (encoded by five genes) will be considered to be the ‘canonical’ histone H2A and will simply be referred to as H2A. Based on the genetic nomenclature, the second most abundant isoform will be referred to as H2A 1C (for HIST**1**H2A**C**). Likewise, the third most abundant isoform will be referred to as H2A 1B/E (Figure 1A).

The replication-dependent histone isoforms are generally thought to be functionally interchangeable. However, in a recent study, the liquid chromatography–mass spectrometry (LC/MS) profile of core histones isolated from B cells from patients with chronic lymphocytic leukemia (CLL) was compared with the profile of histones isolated from CD19+ B cells from healthy individuals. Surprisingly, a decrease in the abundance of H2A 1C and H2A 1B/E was observed in a high percentage of the CLL samples (11). We now report that similar changes in H2A 1C and H2A 1B/E were found in bladder cancer cells. This led us to hypothesize that replication-dependent isoforms can be functionally distinct. We present two lines of evidence to support this hypothesis. First, siRNA knockdown of H2A 1C and H2A 1B/E, but not canonical H2A, led to increased cell proliferation and tumorigenicity. Second, the expression of replication-dependent H2A isoforms can be subject to individualized levels of regulation. The 5′ UTR of the mRNA encoding H2A 1C uniquely imparts translational repression. We also demonstrate that this repression maps to a specific duplicated sequence element found in this 5′ UTR. These observations suggest that replication-dependent histone isoforms can be functionally distinct and play specific roles in the regulation of cell growth.

## MATERIALS AND METHODS

### Cell growth

Human non-invasive (RT4) and invasive (T24) bladder cancer cell lines, and 293TN and U2OS cell lines were purchased from the American Type Culture Collection (ATCC; Manassas, VA). Immortalized (hTERT) and normal human bladder epithelial cells were obtained from Dr Margareta Knowles (St James’s University Hospital, Leeds, UK) and Lonza Walkersville, Inc. (Walkersville, MD), respectively. 293TN and U2OS cells were cultured in Dulbecco’s modified Eagle’s medium (DMEM) (Sigma, St. Louis, MO), and RT4 and T24 cells were cultured in RPMI 1640 medium (GIBCO, Grand Island, NY); all supplemented with 10% fetal bovine serum (Hyclone, Logan, UT), 2 mM l-glutamine, and penicillin (10 U/ml) and streptomycin (10 mg/ml) (GIBCO, Grand Island, NY) and maintained at 37°C in a 5% CO_2_/95% air, humidified atmosphere. hTERT and normal human bladder epithelial cells were grown in Keratinocyte Serum Free Medium (GIBCO, Grand Island, NY) with supplements of bovine pituitary extract and epidermal growth factor plus 30 ng/ml Cholera toxin (Sigma Chemical Company, St. Louis, MO).

### Histone extraction

Cells were seeded, grown for 24–48 h (based on our previous experiences and the growth kinetics of each cell line), harvested by scraping and then snap frozen. Histones were extracted as described previously (12). Briefly, cell pellets were resuspended in 1 ml of NP-40 lysis buffer [10 mM Tris–HCl (pH 7.4), 10 mM NaCl, 3 mM MgCl2, 0.5% NP-40, 0.15 mM spermine, 0.5 mM spermidine, 1 mM phenylmethylsulfonyl fluoride (PMSF), protease inhibitor cocktail (1:1000)] and incubated on ice for 5 min. The nuclei were pelleted at 483*g* for 15 min at 4°C, and the pellet washed with 1 ml of TBS [10 mM Tris–HCl (pH 7.4), 150 mM NaCl]. Sulfuric acid (H_2_SO4) (0.2 M) was added to the washed pellet to extract the histones, and it was vortexed and incubated on ice for 30 min. The solution was centrifuged at 12 045*g* for 15 min at 4°C to remove the cellular debris. Eighty percent acetone was added to the supernatant and precipitated at −20°C overnight. The precipitated histones were centrifuged at 12 045*g* for 15 min at 4°C, allowed to air-dry for 10 min and resuspended in high performance liquid chromatography water.

### LC-MS

Protein concentration was determined by Bradford analysis. Thirty micrograms of purified histones were characterized by LC-MS analysis. LC-MS analysis was performed with a Dionex U3000 high performance liquid chromatography (Dionex; Sunnyvale, CA) coupled to a MicroMass Q-Tof (MicroMass, Whythenshawe, UK). Reversed-phase separation was carried out on a Discovery Bio Wide Pore C18 column (1.0 × 150 mm, 5 µm, 300 Å; Supelco, USA). Mobile phases A and B consisted of water and acetonitrile with 0.05% trifluoroacetic acid, respectively. The flow rate was 25 µl/min, and the gradient started at 20% B, increased linearly to 30% B in 2 min, to 35% B in 8 min, 50% B in 20 min, 60% B in 5 min and 95% B in 1 min. After washing at 95% B for 4 min, the column was equilibrated at 20% B for 30 min, and a blank was run between each sample injection. The cone voltage on the Q-Tof was 25 V. LC-MS data were deconvoluted using MassLynx 4.1.

### Real time RT-PCR

RNA was isolated from the cell lines using Trizol (Invitrogen). The RNA was reverse transcribed using High Capacity cDNA Reverse Transcription Kit (Applied Biosystems), and 50 ng of cDNA was used as template for qPCR reaction. The primers for all the histone isoforms and Taqman mastermix were purchased commercially (Applied Biosystems). The reaction was carried out on an Applied Biosystems 7300 Real Time PCR systems instrument and relative quantitation carried out using 2^−^^ΔΔCt^ method (13).

### siRNA transfection

The siRNAs for each histone isoform were from Applied Biosystems. Transfection was carried out using siPORT NeoFX transfection reagent (Invitrogen) as per manufacturer’s instructions.

### Cell proliferation assay

For each cell type, the initial count was carried out using a hemocytometer, and the cells were plated in 6-well plates in triplicate for every 12 h reading. At each interval, the cells were trypsinized and counted in a Z1 coulter particle counter (Beckman Coulter).

### Soft agar assay

For soft agar assay, 1% base agar was prepared and allowed to cool to 40°C in a water bath and mixed with DMEM, supplemented with 15% fetal bovine serum to make a final agar concentration of 0.5%. Five milliliters of this molten mixture was added in a 100 mm plate and allowed to solidify for 10 min. For preparation of top agarose layer, 1.5% agarose was prepared and mixed 1:1 with DMEM. Each of the siRNA transfected cells and control cells were trypsinized and added to this agar–DMEM mixture to a final agarose concentration of 0.7% and 400 000 cells per plate. The plates were incubated at 37°C, and images were taken after 4–7 days using Zeiss Axioskop Widefield Light Microscope. For quantitative soft agar assay, we used CytoSelect™ 96-Well Cell Transformation Assays kit (Cell Biolabs) as per manufacturer’s instructions.

### Luciferase assays

Oligos corresponding to the 5′ UTR of each of the histone H2A isoforms were designed and cloned upstream of the promoter of the pcDNA-Luc vector. The cloned vectors were then transfected into different cell lines using lipofectamine (Invitrogen). Each of the isoforms was also co-transfected with a Renilla luciferase vector as an internal standard, and the transfection was carried out in triplicates in 6-well plates. After 36 h, the cells were harvested, lysed and luciferase levels estimated using Dual Glo luciferase assay kit (Promega) as per manufacturers protocol. For HuR knockdown, cell lines were treated with siHuR (Santa Cruz Biotechnology) for 6–8 h before transfection with the luciferase vector. Luciferase levels were estimated as described earlier in the text, and one set of cell lines was also used for western analysis using HuR antibody (Santa Cruz Biotechnology).

## RESULTS

### Characterization of the histone H2A complement in bladder cancer cells

To determine whether alterations in replication-dependent histone H2A isoforms is a phenomenon unique to CLL or whether it might be a more common aspect of tumorigenic cells, we analyzed the complement of H2A proteins in a completely unrelated tissue and tumor type. Procedures that had previously been validated were used where histones purified by acid extraction were resolved and analyzed by LC/MS (11). Figure 1B shows the LC/MS profile of histone H2A from normal human bladder epithelial cells, immortalized human bladder epithelial cells (hTERT) and non-invasive (RT4) and invasive (T24) human bladder cancer cell lines. It is clear that there was a significant decrease in the relative abundance of H2A 1C and H2A 1B/E, as the bladder cells acquired the ability to proliferate more vigorously and that there was a further decrease as the cells become tumorigenic. These results suggested that regulation of the abundance of specific replication-dependent H2A isoforms was not limited to CLL and, hence, may be a more general aspect of carcinogenesis.

### Functional analysis of replication-dependent histone H2A isoforms

**Figure 2.**
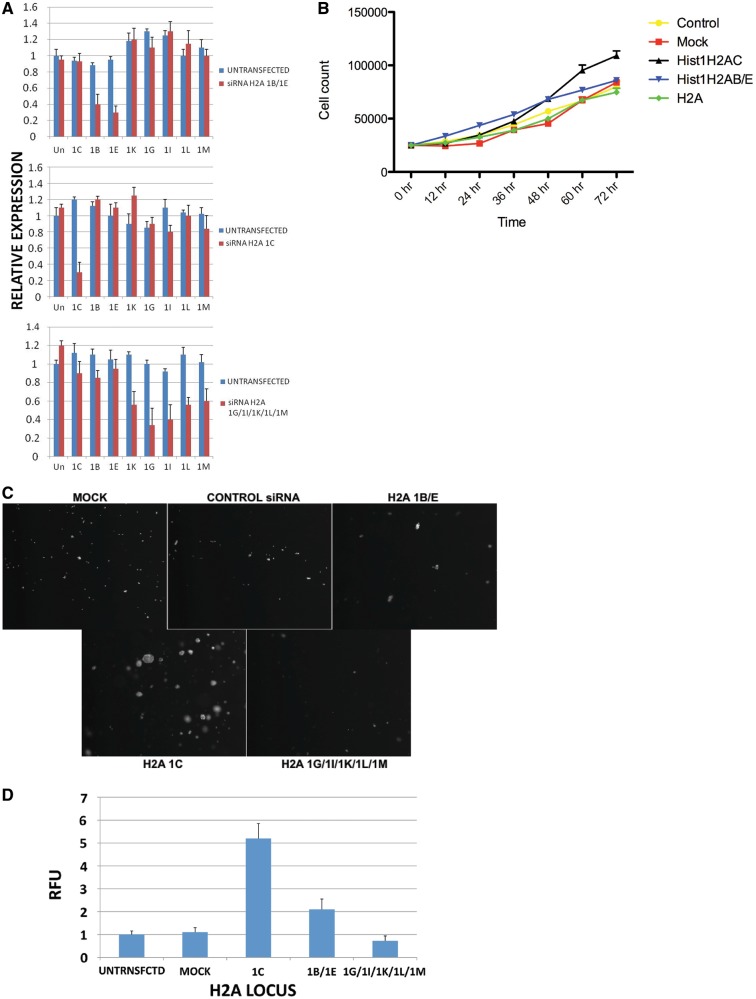
Distinct roles of replication-dependent histone H2A isoforms in cell proliferation and carcinogenesis. (**A**) 293TN cells were transfected with siRNAs targeting the indicated histone H2A loci. qRT-PCR assays were used to measure each H2A mRNA before (blue bars) and after (red bars) transfection. (**B**) The 293TN cells were transfected with the indicated siRNAs (or mock transfected). Cell proliferation was measured by counting cell numbers at the indicated times. (**C**) The 293TN cells were transfected with the indicated siRNAs and plated in soft agar media. Plates were photographed following 5 days of growth. (**D**) The 293TN cells treated with the indicated siRNAs were plated in soft agar in microtiter wells. Following 6 days, cell growth was quantitated by solubilizing the cells in the presence of Cytoselect (Cytoselect 96-well cell transformation assay kit, Cell Biolabs, Inc.) and measuring relative fluorescence intensity.

To determine whether replication-dependent histone H2A isoforms are functionally distinct, we used an siRNA strategy to downregulate the H2A isoforms and determine whether a decrease in specific isoforms leads to distinct effects on cell growth and proliferation. The 293TN cells were transfected with either control siRNAs, a mixture of siRNAs that target all five genes that encode canonical H2A, a pair of siRNAs that target the H2A 1B/E genes or an siRNA targeting the H2A 1C gene. The specificity of the siRNA knockdown was confirmed using real time PCR assays. As seen in Figure 2A, the abundance of only the targeted mRNAs was specifically decreased.

The effect of the siRNA knockdowns on the rate of cell proliferation was tested by measuring the growth rate of cells treated with each of the siRNAs. As seen in Figure 2B, siRNA knockdown of the canonical H2A genes had no effect on cell proliferation, knock down of H2A 1B/E caused a modest increase in cell proliferation and siRNA knockdown of H2A 1C resulted in a marked increase in the rate of cell proliferation.

To determine whether the siRNA knockdown of the H2A isoforms had an impact on cell tumorigenicity, soft agar assays were performed following the siRNA treatment of cells. Strikingly, the knockdown of H2A 1C induced the formation of large colonies (Figure 2C). Knockdown of H2A 1B/E resulted in a small increase in colony formation, whereas depletion of canonical H2A had a slightly negative impact on colony formation. The effect of H2A isoform knockdown on cell proliferation in soft agar was also quantified using a fluorescent dye-based assay with similar results observed (Figure 2D).

**Figure 3.**
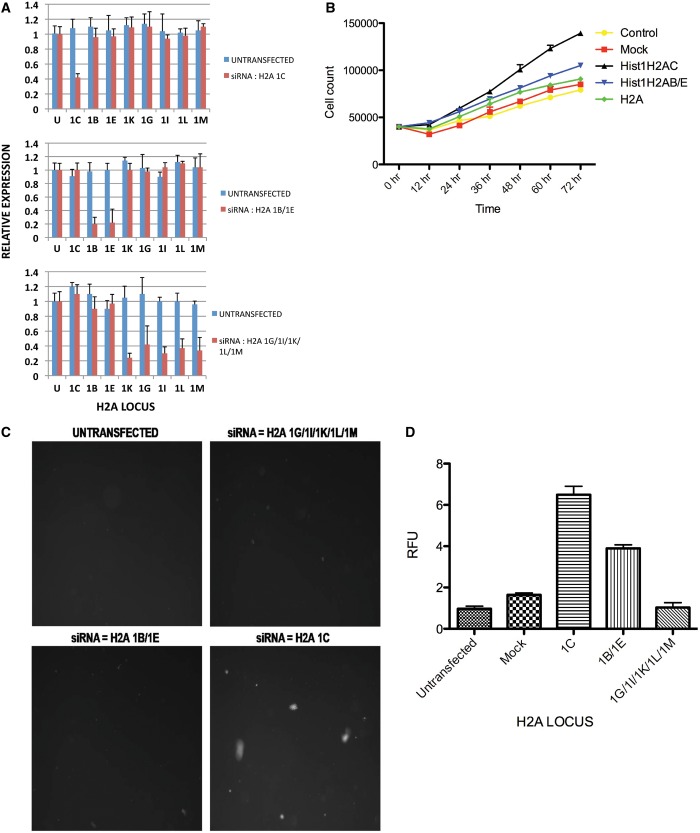
Histone H2A 1C influences cell proliferation and carcinogenesis in U2OS cells. (**A**) U2OS cells were transfected with siRNAs targeting the indicated histone H2A loci. qRT pcr assays were used to measure each H2A mRNA before (blue bars) and after (red bars) transfection. (**B**) Cells were transfected with the indicated siRNAs (or mock transfected). U2OS cell proliferation was measured by counting cell numbers at the indicated times. (**C**) U2OS cells were transfected with the indicated siRNAs and plated in soft agar media. Plates were photographed following 5 days of growth. (**D**) U2OS cells treated with the indicated siRNAs were plated in soft agar in mitrotiter wells. Following 6 days, cell growth was quantitated by solubilizing the cells in the presence of Cytoselect (Cytoselect 96-well cell transformation assay kit, Cell Biolabs, Inc.) and measuring relative fluorescence intensity.

To determine whether the effect of H2A isoform modulation on cell proliferation and tumorigenicity was cell-type specific, we repeated the analyses using a different cell line. As seen in Figure 3A, specific siRNA-based depletion of H2A isoforms was also achieved in U2OS cells. Strikingly, the effect of this H2A isoform knockdown on cell proliferation and growth in soft agar was similar to that seen with 293TN cells. Namely, siRNA knockdown of H2A 1C caused a significant increase in cell growth and a dramatic increase in the colony size observed during growth on soft agar, whereas siRNA knockdown of H2A 1B/E has a smaller effect on both phenotypes (Figure 3B–D).

If replication-dependent histone H2A isoforms are functionally equivalent, depletion of any of the isoforms would be predicted to have a similar effect on cell proliferation. Our results clearly show that this is not the case and that knockdown of specific isoforms can have different effects on cellular phenotypes.

### Regulation of replication-dependent histone H2A isoform gene expression

The genes encoding the replication-dependent histone isoforms are grouped together in several large clusters. These histone clusters localize to Cajal bodies in the nucleus, which are thought to be the sites at which the histone genes are transcribed. Based on the clustering and co-localization of the histone genes, it is presumed that they are transcriptionally regulated as a unit through common mechanisms. However, if replication-dependent isoforms, such as H2A 1C and H2A 1B/E, have distinct cellular functions, it would be predicted that there might be isoform-specific gene regulatory mechanisms. Therefore, we have begun to analyze the expression of these genes at the mRNA level.

**Figure 4.**
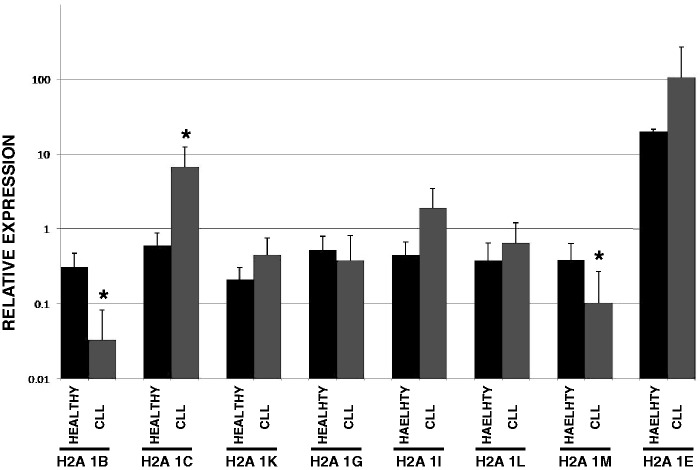
Replication-dependent histone H2A gene expression in healthy individuals and CLL patients. Real time PCR assays were used to measure the abundance of the indicated histone H2A genes in either healthy individuals or CLL patients (N = 129). The relative expression of each isoform was determined by normalizing the PCR reactions to a control reaction that contained an equal amount of each H2A target cloned into a vector (50 fg each). H2A isoforms showing a significant change in abundance in CLL patients (*P* < 0.01) are indicated by an asterisk.

Total RNA was isolated from CD19+ B cells selected from healthy volunteers. In addition, RNA was isolated from the B cells of >100 CLL patients. Real time PCR assays were used to measure mRNA levels for the eight replication-dependent H2A genes that encode the canonical H2A, H2A 1C and H2A 1B/E (Figure 4). In addition to an internal control (PGK1), the PCR reactions were also normalized to a control reaction that contained equal quantities of each H2A gene so that the absolute abundance of the mRNAs could be compared. In cells from the healthy individuals, most of the H2A isoform mRNAs are found at similar levels. The striking exception is HIST1H2AE, which is present at levels that are ∼20-fold higher than the other isoforms. Also, although most of the replication-dependent H2A isoforms did not significantly change in the CLL patient cells, three of the isoforms showed significant changes in abundance in these tumor cells. The HIST1H2AB and HIST1H2AM mRNAs were downregulated in CLL and the HIST1H2AC mRNA was upregulated. These results indicate that individual replication-dependent histone H2A isoform genes can be subject to individual regulation at the mRNA level.

Replication-dependent H2A isoforms may also be subject to posttranscriptional regulation. To explore this idea, we have begun to dissect the H2A transcripts to identify functional regulatory elements. As would be expected from the high level of protein sequence conservation, the coding sequences of the eight histone H2A genes that encode the majority of H2A isoforms are nearly identical. Outside of the coding sequences, there are two blocks of sequence variation that could potentially provide platforms for regulation. The first is the 5′ UTR and the second is a short stretch of the 3′ UTR between the stop codon and the invariant stem–loop.

**Figure 5.**
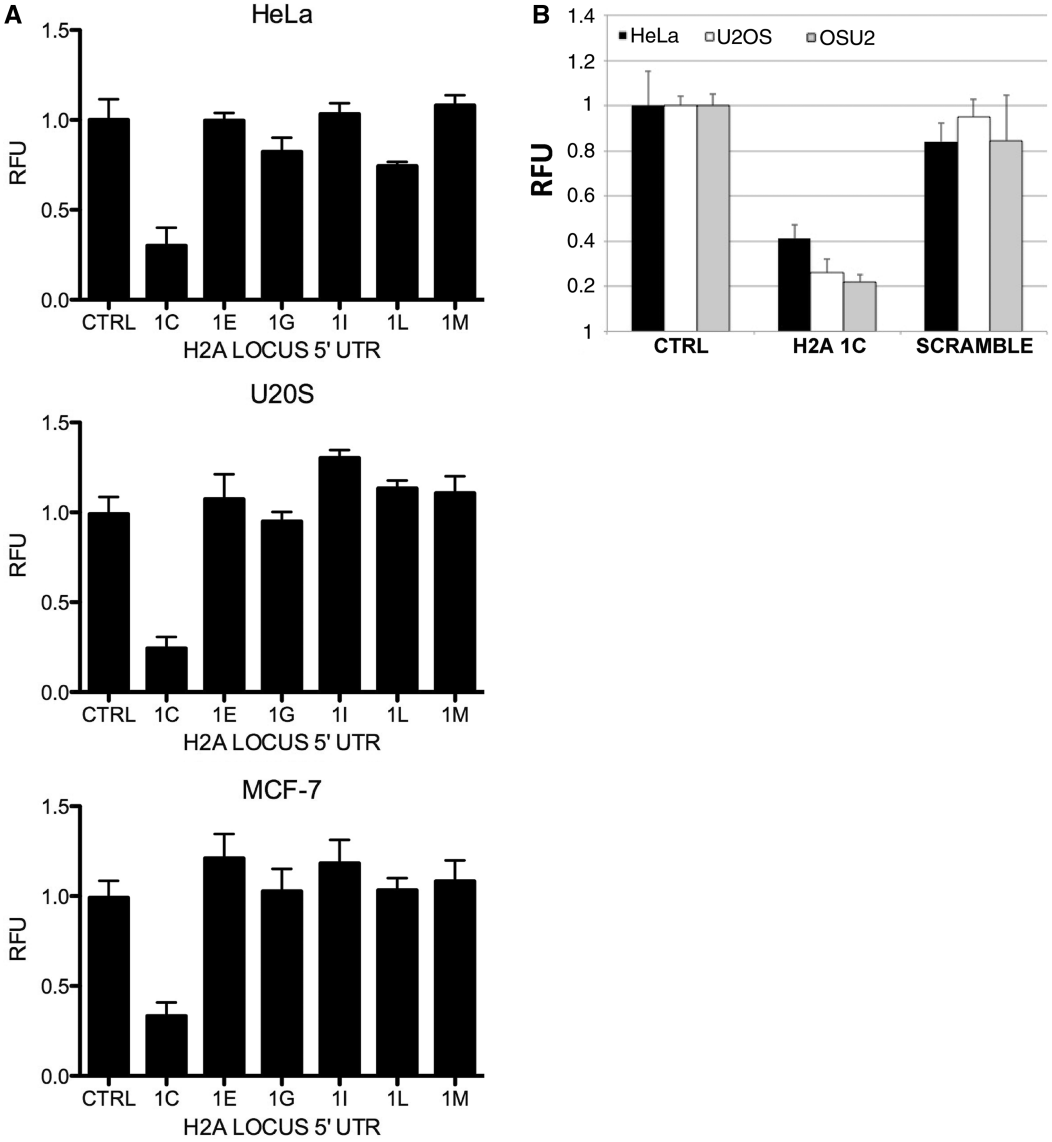
The 5′ UTR of HIST1H2AC is a regulatory element. (**A**) The 5′ UTR of each of the indicated H2A isoforms was cloned upstream of a luciferase reporter construct. Each construct was transfected into the indicated cell lines (with a renilla reporter for normalization) and luciferase levels determined by fluorescence. (**B**) Luciferase levels were determined as in (A) using constructs that contained either the wild type HIST1H2AC 5′ UTR (1C) or a scrambled version that contains the same base composition but in a random order (scramble).

To determine whether the 5′ UTRs of the replication-dependent histone H2A genes influence gene expression, each 5′ UTR was cloned directly upstream of the coding sequence of a luciferase reporter construct. Each vector was transfected into cells (along with a renilla reporter plasmid for normalization), and the luciferase levels were determined by fluorescence intensity. As seen in Figure 5A, the 5′ UTR of the HIST1H2AC locus was unique in its ability to alter luciferase expression (the mRNA for two of the H2A isoforms have no 5′ UTR). In multiple cell lines, the HIST1H2AC 5′ UTR caused a significant downregulation of luciferase (typically 3- to 5-fold), suggesting that this sequence could mediate translational repression. Importantly, a scrambled version of the HIST1H2AC 5′ UTR, which contains the same base composition but with a randomized sequence, did not alter expression when fused to luciferase (Figure 5B). Taken together, these results demonstrate that replication-dependent histone H2A genes are subject to individualized regulation at the transcriptional and posttranscriptional levels.

### Analysis of the HIST1H2AC 5′ UTR

**Figure 6.**
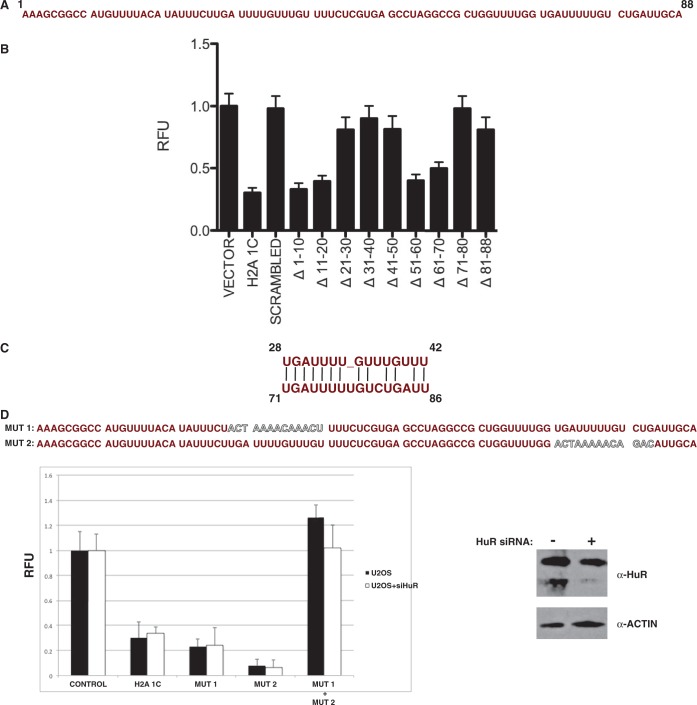
Identification of a repeated element in the H2A 1C 5′ UTR necessary for repressive activity. (**A**) Sequence of the H2A 1C 5′ UTR separated into 10 base segments. (**B**) Luciferase constructs containing the indicated H2A 1C 5′ UTR sequences were transfected into U2OS cells and assayed for fluorescence intensity. Control contains no insert, whereas the H2A 1C contains the entire H2A 1C 5′ UTR. The scrambled construct contains a randomized version of the H2A 1C sequence. The remaining constructs contain a deletion of the indicated bases from the H2A 1C 5′ UTR. The fluorescence signal of the control construct was set to 1 for each cell line. (**C**) Highly similar sequence elements identified in the indicated regions of the H2A 1C 5′ UTR. (**D**) Sequences of the H2A 1C 5′ UTR containing either the MUT1 or MUT2 mutation (top, altered sequences shown in outlined text). Lucifierase constructs containing the indicated H2A 1C 5′ UTR sequences were transfected into U2OS cells and assayed for fluorescence intensity (bottom left). Aliquots of cells were also treated with an siRNA directed against HuR 48 before luciferase assay. Efficiency of HuR knockdown was assayed by western blot analysis of whole-cell extracts using the indicated antibodies (bottom right).

To determine whether the HIST1H2AC 5′ UTR contains discrete sequences that mediate its regulatory activity; we performed a deletion analysis of this element. Ten base deletions were made across the length of the 5′ UTR in the context of the luciferase fusion (Figure 6A). As seen in Figure 6B, two sets of contiguous deletions resulted in a loss of translational repression. This suggested that two regions of the 5′ UTR were important for its regulatory activity. These regions span bases 21–50 and 71–88. Conversely, deletion of bases 1–20 and 51–70 had little impact on the repression mediated by the HIST1H2AC 5′ UTR. Importantly, identical results were observed in multiple cell lines (Figure 6B).

Comparison of the sequences between 21 and 50 and between 71 and 88 of the HIST1H2AC 5′ UTR indicated that they each region contained a highly similar 15/16 base sequence (Figure 6C). These sequences span bases 28–42 and 71–86 of the HIST1H2AC 5′ UTR and are identical at 13 positions and the position of these sequences is entirely consistent with the results of the deletion analysis. To more precisely probe the functional significance of this sequence, mutations were constructed that altered the sequence of either the first element (MUT1, Figure 6D), the second element (MUT2, Figure 6D) or both elements (MUT1 + MUT2). As shown in Figure 4D, neither the MUT1 nor MUT2 construct showed any decrease in repression. However, mutating both sites caused a complete loss of translational repression. These results indicate that this conserved sequence plays a critical role in the posttranscriptional regulation of the HIST1H2AC gene.

The most striking aspect of this conserved sequence element is that it contains poly U stretches. In many contexts, this type of poly U stretch has been found to be a binding site for the HuR protein (14,15). Although HuR binding sites are predominantly found in the 3′ UTR of HuR-regulated genes, there are also a significant number of mRNAs that contain HuR-binding sites in their 5′ UTR (16,17). Genomic analyses of HuR target mRNAs detected binding of HuR to the 5′ UTR of the HIST1H2AC mRNA, and, notably, HIST1H2AC was the only replication-dependent isoform of H2A associated with HuR (17). Intriguingly, 5′ UTR HuR binding has been shown to mediate translational repression (18–20). Therefore, we sought to determine whether HuR played a role in the regulation mediated by the HIST1H2AC 5′ UTR. However, knockdown of HuR protein levels by siRNA treatment had no effect on the translational repression mediated by the HIST1H2AC 5′ UTR, indicating that the conserved sequence element identified in this regulatory region does not function solely through HuR.

## DISCUSSION

Although the important role of histone variants in the regulation of chromatin structure has been firmly established, a functional role for the primary sequence diversity represented by the replication-dependent families of histone isoforms has not been addressed. Taken together, our results provide several lines of evidence that support the hypothesis that replication-dependent histone H2A isoforms are functionally distinct proteins. First, the relative abundance of specific replication-dependent isoforms varied between normal tissue samples and tumor tissue samples at both the protein and mRNA levels. Second, artificial manipulation of replication-dependent H2A isoform levels by siRNA knockdown resulted in changes in the rates of cell proliferation and tumorigenicity *in vitro*. Third, replication-dependent H2A isoforms can be subject to individualized mechanisms of regulation. This is exemplified by the presence of specific elements in the 5′ UTR of HIST1H2AC mRNA that mediate translational repression.

**Figure 7.**
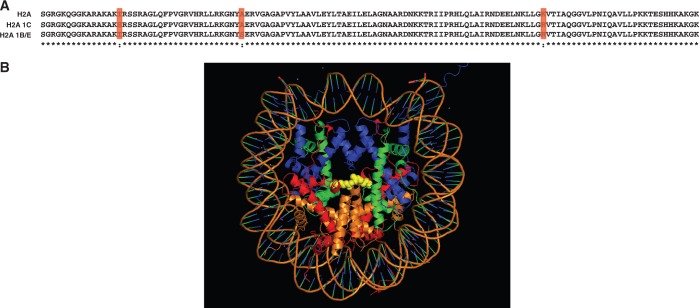
H2A 1C and H2A 1B/E share a common amino acid change. (**A**) Protein sequence alignment of H2A, H2A 1C and H2A 1B/E. Positions of identity are marked with an asterisk. Positions of divergence are highlighted in red. (**B**) The crystal structure of the nucleosome with residue 99 of histone H2A shown in yellow as a space-filling model.

The idea that the replication-dependent histone isoforms are functionally interchangeable stems largely from the high degree of sequence identity found between the isoforms. H2A 1C and H2A 1B/E each differ from canonical H2A at two positions (Figure 7A). The threonine at position 16 is changed to serine in H2A 1C and the alanine at residue 40 is changed to serine in H2A 1B/E. In addition, the second change is shared by both H2A 1C and H2A 1B/E. This change is at position 99 where canonical H2A contains a lysine residue and H2A 1C and H2A 1B/E each contain an arginine residue. Interestingly, the presence of a lysine at residue 99 in canonical H2A is seen in many primate species, whereas in most mammals, including mice, the canonical H2A contains an arginine at this position (21). As depicted in Figure 7B, this residue is located in the center of the nucleosome face. Although changing lysine to arginine is considered to be a conservative substitution in most proteins, the identities of these residues in the core histones are highly conserved throughout eukaryotic evolution (1). This may be due to the important role that these residues play in the regulation of chromatin structure as sites of post-translational modification. Lysine 99 has recently been identified as a site of methylation on histone H2A (22). In addition, the other changes in H2A 1C and H2A 1B/E either eliminate or introduce a potential site of phosphorylation. Hence, although the number of amino acid changes in H2A 1C and H2A 1B/E are small, we hypothesize that these differences have the potential to confer distinct functions to these isoforms.

The presence of multiple replication-dependent histone isoform genes in large clusters is likely to serve a number of purposes. Given the large amount of histone proteins needed to assemble newly replicated DNA into chromatin, it is likely that the output of multiple histone genes is required to produce sufficient quantities of histone protein. In addition, the proximity of the replication-dependent histone genes facilitates their clustering near Cajal bodies allowing for the coordinate regulation of this important gene family (7). Given the high degree of sequence identity between the replication-dependent histone genes, techniques such as northern blotting would only be able to look at the bulk levels of histone mRNAs and would not be able to discriminate between the expression of distinct genes. Our results suggest that expression of the different replication-dependent histone genes within the same cluster is not uniform. For example, transcripts from the HISTH2AE gene are far more abundant than the other H2A genes. In addition, the levels of some, but not all, replication-dependent H2A isoform genes can be significantly up- or downregulated in tumor tissue samples. Whether these effects are the result of transcriptional or posttranscriptional regulation of histone gene expression will be important to determine.

Histone mRNA 3′-end formation has clearly been shown to be the critical factor in the regulation of histone gene expression (7). However, our results suggest that the 5′ UTR of replication-dependent histone genes can also play a regulatory role. Although the 3′ stem–loop structure is ubiquitously present in replication-dependent histone genes, the regulatory elements identified in the 5′ UTR of the HIST1H2AC mRNA appear to be gene-specific. Like many RNA regulatory elements, the 5′ UTR of HIST1H2AC contains a high concentration of AU- and GU-rich sequences. Although the well-characterized ARE binding protein HuR does not appear to be involved in the HISTH2AC 5′ UTR, there are many proteins that have been shown to interact with similar sequences that are now candidate regulators of this element. The identification of this regulatory element may help to identify novel regulators of histone gene expression.

## FUNDING

National Institutes of Health [P01 CA101956 to M.R.P. and J.C.B. and CA107106 to M.A.F.]; a Specialized Center of Research award from the Leukemia and Lymphoma Society (to J.C.B.); and The Ohio State University Comprehensive Cancer Center Molecular Carcinogenesis and Chemoprevention Program (to S.K.C. and A.M.). Funding for open access charge: P01 CA101956 R01 CA107106 Specialized Center of Research award from the Leukemia and Lymphoma Society.

*Conflict of interest statement*. None declared.
